# Supplementary material for: NpPP2-B10, an F-Box-Nictaba Gene, Promotes Plant Growth and Resistance to Black Shank Disease Incited by Phytophthora nicotianae in Nicotiana tabacum
Source: Int J Mol Sci. 2023 Apr 16;24(8):7353. doi: 10.3390/ijms24087353 (PMC10138871; doi:10.3390/ijms24087353)
Supplement: Supplementary file 1 [file ijms-24-07353-s001.zip › Supplementary Materials.pdf]

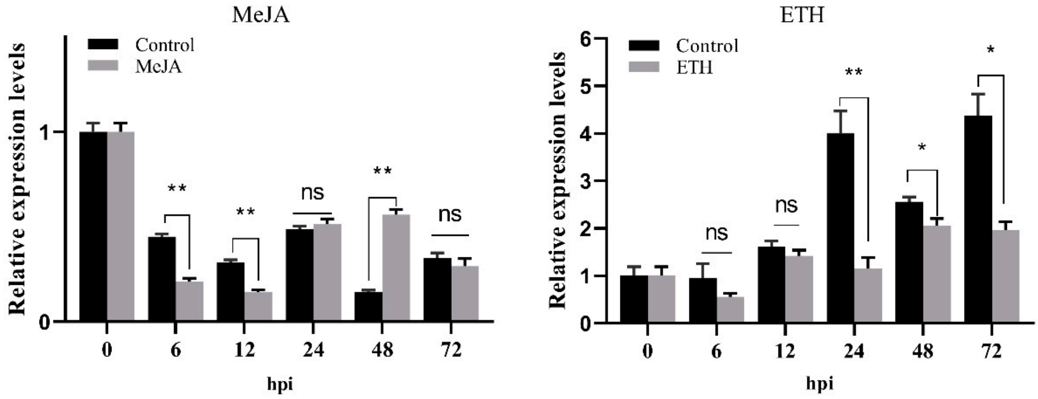


**Figure S1.** Expression pattern of the c62451.graph_c0 gene after treatment with MeJA and ETH. Asterisks denote significant differences, * $p < 0.05$, ** $p < 0.01$, by Student's t-test; ns, no significant difference.

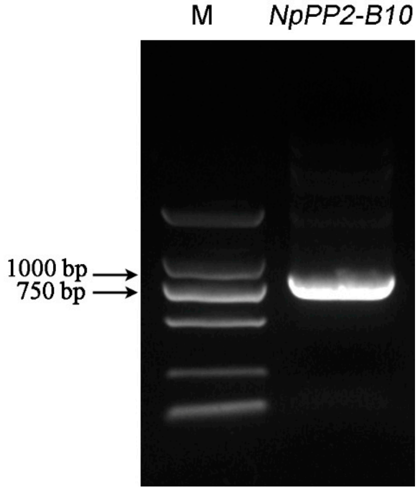


**Figure S2.** Electrophoretic map of the *NpPP2-B10* gene clone.

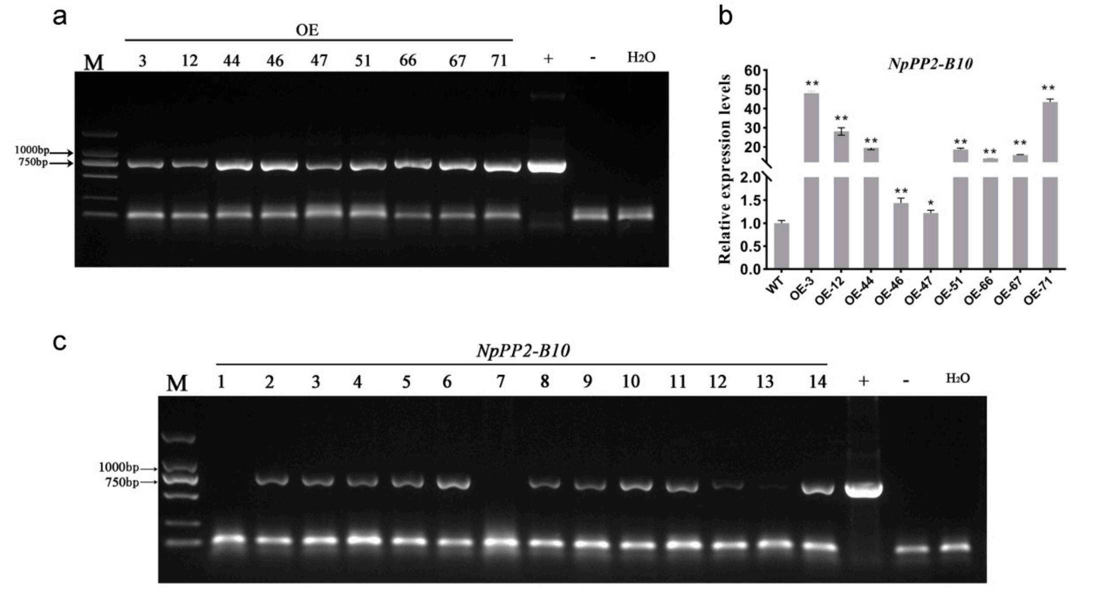


**Figure S3.** Identification of *NpPP2-B10*-positive plants in T0 and T1 generations. (a), *NpPP2-B10* gene T0 generation positive plants; (b), Relative expression level of *NpPP2-B10* overexpressed plants in T0 generation; Number of samples, n = 3; Asterisks denote significant differences, * $p$<0.05, ** $p$<0.01; (c), T1-positive plants of *NpPP2-B10*; +, 35S::*NpFBA1* recombinant plasmid was used as the positive control; -, Negative control using WT genomic DNA as negative control; $H_2O$, Blank control with water as template.

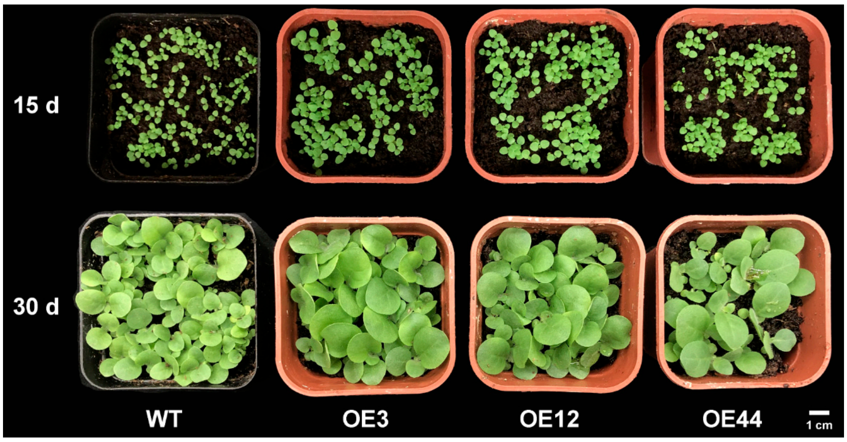


**Figure S4.** *NpPP2-B10* overexpressed lines and WT seedling growth.

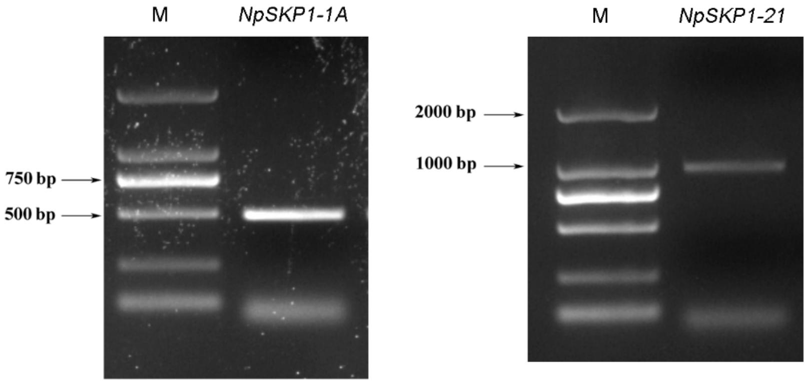


**Figure S5.** Electrophoretic map of *NpSKP1-1A* and *NpSKP1-21* gene clones.

MKSYIWLQTADGSIQQVEEEVAMFCPMICREVLQTGTGSSKNCAISLPQRVNAAILGLILDYCR

FHQVPGRSNKERKTFDEKFIRLDTKKLCELTSAADSLQLRPLVDLTSRALARMIEGKTPEEIRETF

SKP1

HLPDDLTEEEKLEPLRNMTDGPRIRLLNRLHARKRKELKEREKLKNVEVEEEQHVDERSVDDLL

SFINGEDEDSKGVRTTKSKKKNRRRKEQARNSSTANETSSHNQESSFPASSCLNGDVPSPSKPS

DLQDPAFDEGDIDDELDPVMKEEIDREVEDFARRLNSVWPERMQEILSLGQERRPVPLSVNG

NGSLKRYTAGLDGR-

**Figure S6.** Amino acid sequence of NpSKP1-21.

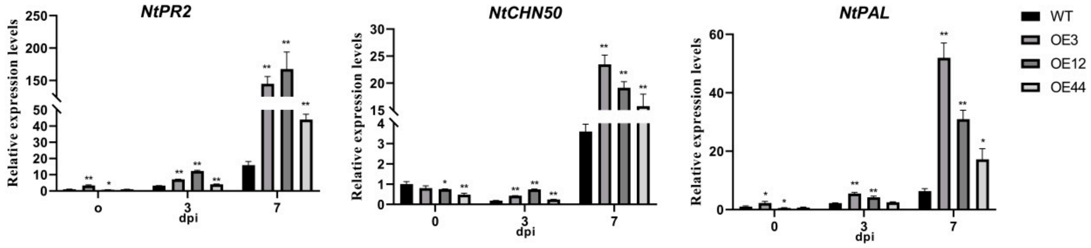


**Figure S7.** Expression levels of WT disease-resistant genes in *NpPP2-B10* overexpressed lines after infection with *P. nicotianae* race 0. Number of samples, n = 3; Asterisks denote significant differences, * $p<0.05$, ** $p<0.01$.

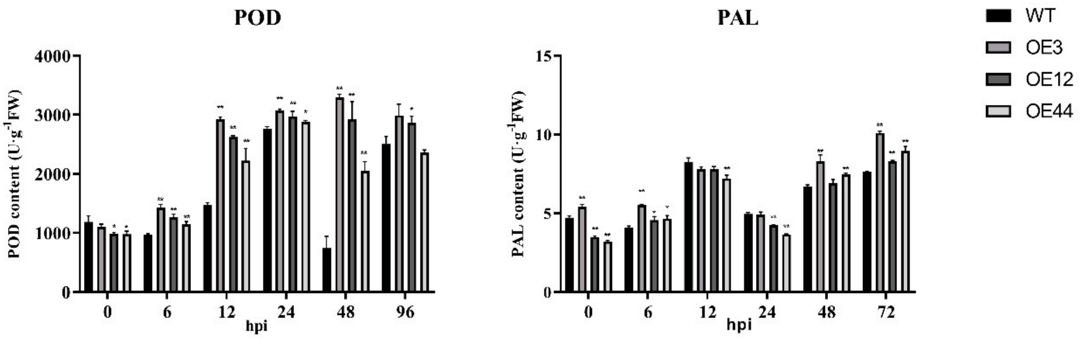


**Figure S8.** POD and PAL enzyme activities of *NpPP2-B10* overexpressed lines and WT after infection with *P. nicotianae* race 0. Number of samples, n = 3; Asterisks denote significant differences, * $p < 0.05$, ** $p < 0.01$.

**Table S1.** Primers used for quantitative real−time PCR (qRT–PCR).

| Primer name | Forward primer sequences | Reverse primer sequences |
|---|---|---|
| *NpPP2-B10* | GACGGTTGCGATTCCATA | GCCTCACTTCTCCGTATTG |
| *NpActin* | TGAGATGCACCACGAAGCTC | CCAACATTGTCACCAGGAAGTG |
| *NtPR1* | AACCCATCCATACTATTCCTTG | GCCGCTAACCTATTGTCCC |
| *NtPR2* | GCTCCTGCCATGCAAAATGT | ATCTTTGGGCGGGTTGGTAT |
| *NtPAL* | GCAGGGCATTCGGTTTGAGA | GGACTGGACAGCATCCAACA |
| *NtCHN50* | ATGCCAAGGAAAGGGATTCTACA | TGGGAGGTTTGGGCGAAGA |
| *NtActin* | TGAGATGCACCACGAAGCTC | CCAACATTGTCACCAGGAAGTG |

**Table S2.** Primers used for vector construction.

| Primer name | Forward primer sequences（5′-3′） | Reverse primer sequences（5′-3′） |
|---|---|---|
| pCAMBIA2300- *NpPP2-B10* | ggacagggtacccggggatccATGGATTATTTTCGTTTATTACCAGAAGG | caggtcgactctagaggatccTCATTCTGGTCGAAACTCCATTC |
| pCAMBIA2300-eGFP-*NpPP2-B10* | ggacagggtacccggggatccATGGATTATTTTCGTTTATTACCAGAAGG | agtgtcgactctagaggatccTTCTGGTCGAAACTCCATTC |
| pETSUMO-*NpPP2-B10* | ggtggtggatccgaattccggactATGGATTATTTTCGTTTATTAC | ggtggtggtgctcgagtgcggccttaTCATTCTGGTCGAAACTCCATTC |
| pGADT7 -*NpSKP1-1A* | catatggccatggaggccagtgaattcATGTCTACTTCAAAAATGATTGTGTTGAAG | catctgcagctcgagctcgatggatccTCACTCAAAGGCCCACGCAT |
| pGADT7 -*NpSKP1-21* | catatggccatggaggccagtgaattcATGAAGTCCTACATTTGGCTCCAAA | tggatcccgtatcgatgcccacccgggTTATCTCCCATCCAAACCTGCTGTA |
| pGBKT7 - *NpPP2-B10* | ctgcatatggccatggaggccgaattcATGGATTATTTTCGTTTATTACCAGAAGG | atgcggccgctgcaggtcgacggatccTCATTCTGGTCGAAACTCCATTC |
| pSPYCE- *NpSKP1-1A* | caggcctggcgcgccactagtggatccATGTCTACTTCAAAAATGATTGTGTTGAAG | gtacatcccgggagcggtaccctcgagCTCAAAGGCCCACGCAT |
| pSPYNE - *NpPP2-B10* | tttggagagaacacgggggactctagaATGGATTATTTTCGTTTATTACCAGAAGG | gaggtcgacagtactatcgatggatccTTCTGGTCGAAACTCCATTC |
